# Osteoporosis Diagnostics in Dental Radiology (Panoramic Radiograph): The Mini Osteoporosis Pre-Screening (MOPS)

**DOI:** 10.3390/diagnostics16111728

**Published:** 2026-06-04

**Authors:** Margrit-Ann Geibel, Amina Maria Geibel, Meinrad Beer, Tilmann Blasenbrey, Daniela Kildal

**Affiliations:** 1 Dento-Maxillofacial Radiology, Department of Maxillofacial Surgery, University Hospital Ulm, 89070 Ulm, Germany; 2 Department Genderspecific Dentistry, Danube University, 3500 Krems an der Donau, Austria; 3 Department of Oral Surgery, University Center for Dental Medicine Basel UZB, 4058 Basel, Switzerland; amina.geibel@uzb.ch; 4 Department of Diagnostic and Interventional Radiology, University Hospital Ulm, 89070 Ulm, Germany; meinrad.beer@uniklinik-ulm.de (M.B.); daniela.kildal@hopitalvs.ch (D.K.); 5 Radiology, Upper Valais Hospital Center (SZO), Hôpital du Valais, 3900 Brig, Switzerland

**Keywords:** dental radiology, orthopantomogram, panoramic radiograph, cervical spine, osteoporosis, pre-screening

## Abstract

**Background/Objectives:** Osteoporosis is highly relevant to dentistry, oral surgery, and implantology. Because panoramic radiographs are routinely acquired in dental practice, they may enable opportunistic pre-screening. This pilot study evaluated established radiomorphometric indices on orthopantomograms (OPGs) and introduced a new qualitative index, the Mini Osteoporosis Pre-Screening (MOPS) index, based on the appearance of the cervical vertebral bodies visible on panoramic radiographs. **Methods:** This retrospective study analyzed 40 pseudonymized patient datasets, each including both OPG and dual-energy X-ray absorptiometry (DXA) examinations. DXA findings served as the reference standard in the study group. In addition 80 OPG scans from a control group of young adults without clinical or anamnestic evidence of osteoporosis were assessed. Two independent examiners with backgrounds in radiology and dentistry evaluated four indices: mandibular cortical width (MCW), panoramic mandibular index (PMI), mandibular cortical index (MCI), and MOPS. **Results:** Relative to the reference standard, MOPS demonstrated diagnostic accuracy ranging from 83% to 100% across selected groups. In women, accuracy ranged from 90% to 100%, with a sensitivity of 1.0 and specificity of 0.9. MCI and MOPS were more robust than MCW, whereas PMI showed limited applicability in routine clinical images. **Conclusions:** Panoramic radiographs can show osteoporosis-related changes in the mandible and cervical spine. MOPS offers a rapid, cost-neutral pre-screening method and may help dentists identify patients who warrant DXA referral. Larger prospective studies are needed to confirm its clinical utility.

## 1. Introduction

Osteoporosis is a highly prevalent systemic skeletal disease [1]. It is also highly relevant to dentistry, because dentists are likely to encounter patients with osteoporosis routinely. Osteoporosis affects the jaws and teeth, and associations with oral conditions such as caries, periodontitis, and tooth loss have been reported [2,3]. A systematic review demonstrated a correlation between osteoporosis and periodontitis [4]. Bone density is also significantly correlated with the number of remaining teeth. Osteoporosis leads to bone loss in the alveolar processes of the maxilla mandible, thereby reducing bony support for tooth anchorage. Consequently, osteoporosis can contribute to tooth loss, and tooth loss may also serve as a surrogate marker for osteoporosis [2,5,6,7]. Osteoporosis also adversely affects osseointegration and dental implant survival. A systematic review reported impaired implant osseointegration outcomes in patients with osteoporosis [8]. Significant peri-implant bone loss has been observed in these patients. Dental implants remain a viable treatment option for patients with osteoporosis. However, close monitoring by informed specialists and regular assessment of the peri-implant bone stability are required [9]. Because of their frequent patient contact and the large number of radiographs they acquire, dentists are uniquely positioned to contribute to the early diagnosis of osteoporosis.

According to the Robert Koch Institute (2017), the prevalence of osteoporosis was 4.4% in women and 1.9% in men aged 45–64 years. After the age of 65 years, prevalence increases to 24% in women and 6% in men [1]. Osteoporosis often becomes clinically evident only after fractures occur, with approximately 200,000 new vertebral or proximal femoral fractures each year attributable to deteriorating bone mass and quality [10]. Such fractures substantially increase mortality: after a hip fracture, three-month mortality increases five- to eightfold, reaching up to 31%, with a greater and more persistent increase in men [11,12]. The economic burden is considerable, estimated at USD 5000–6500 across Canada, Europe, and the United States, excluding indirect costs such as disability and lost productivity [13]. These costs are projected to increase by approximately 50% between 2010 and 2030 [14], emphasizing the importance of early detection and treatment [14,15]. In the United States, approximately 68% of individuals visit a dentist annually, compared with only 15% who consult a general practitioner [16]. If dentists identified and referred patients with suspected osteoporosis before fractures occur, the preventive benefit could be substantial. Early detection enables timely therapy, potentially preventing fractures and reducing healthcare costs [17].

The current diagnostic standard for confirming osteoporosis is dual-energy X-ray absorptiometry (DXA), which measures bone mineral density (BMD) at the lumbar spine and/or femur [18,19]. Osteoporosis is defined as a BMD T-score at least 2.5 standard deviations below the mean value for healthy premenopausal women [18,19,20,21,22]. In clinical practice, suspicion of osteoporosis may also arise from medical history or screening tools such as the IOF risk test. Notably, approximately 40% of all medical X-ray examinations are performed in dentistry, including orthodontics, and nearly 25% of dental X-rays are single-exposure orthopantomograms (OPGs) [23]. These images may provide an opportunity to identify patients with potentially reduced bone status and refer them for diagnostic assessment before fractures occur. Osteoporosis has typical radiographic characteristics, such as trabecular and cortical thinning in long bones [24]. As a systemic condition, it causes similar changes in the maxilla, mandible, and teeth. Common findings include reduced jawbone mass, altered trabecular pattern, cortical thinning, decreased bone density, loss of alveolar process height, and tooth loss.

Several dental radiology indices can indicate osteoporosis on OPGs. Mandibular cortical width (MCW) is the width of the mandibular cortex at the inferior border, measured perpendicularly below the mental foramen [25,26,27]. The panoramic mandibular index (PMI) is the ratio of MCW to the distance from the mandibular border to the lower margin of the mental foramen [28,29]. The mandibular cortical index, or Klemetti Index (MCI), visually assesses the transition from cortical to cancellous bone at the inferior mandibular border distal to the mental foramen bilaterally [29]. Although these indices can suggest low bone status, their use in everyday dental practice is limited because they are considered time-consuming, technique-sensitive, or impractical. This highlights the need for a simple, robust, and pragmatic OPG-based pre-screening method that is applicable under routine dental imaging conditions. To address this need, the present study introduces Mini Osteoporosis Pre-Screening (MOPS), a qualitative index designed for rapid OPG assessment. The primary aim was to evaluate the applicability and exploratory diagnostic performance of MOPS for identifying DXA-confirmed reduced bone status. Secondary aims were to compare MOPS with established indices, including MCW, PMI, and MCI, and to assess normal classification rates in a young-adult control group and osteoporosis detection rates in a DXA-assessed study group.

## 2. Materials and Methods

### 2.1. Study Design and Hypothesis

This study was designed as a retrospective, single-center, exploratory study of diagnostic accuracy and feasibility. It used pseudonymized imaging data from the institutional archive covering the period from 2015 to 2025. The aim was to determine whether osteoporosis-related radiographic changes can be identified on routine panoramic radiographs and whether such findings could support opportunistic osteoporosis screening in dental practice. No OPGs or DXA examinations were acquired specifically for study purposes. The design therefore reflects a real-world opportunistic screening scenario rather than a controlled diagnostic-validation setting. The reporting of this study was guided by the STARD principles for diagnostic accuracy studies and, because of the retrospective observational design, by applicable STROBE items.

The primary hypothesis was that routine OPGs contain clinically relevant information on systemic bone status. Osteoporosis-related changes may be detectable not only in the mandible but also in the C2 and C3 cervical vertebral bodies, which are visible on panoramic radiographs. Specifically, we hypothesized that the Mini Osteoporosis Pre-Screening (MOPS) index would offer greater practical applicability and a more balanced screening performance than established mandibular radiomorphometric indices in routine clinical imaging settings.

### 2.2. Study Population and Reference Standard

Two groups were analyzed. The study group comprised 40 patients who had undergone both OPG and DXA examinations. In this group, DXA served as the reference standard for reduced bone status. The control group included 80 young adults aged 20–30 years without clinical or anamnestic evidence of osteoporosis and with OPGs suitable for evaluating the mandible and cervical vertebral bodies.

The control group was deliberately selected to represent the age range of expected peak bone mass. This approach is directly aligned with the WHO T-score concept, in which an individual patient’s BMD is interpreted relative to the mean BMD of a healthy young-adult reference population. Therefore, healthy young adults without known risk factors constitute a biologically plausible reference group for evaluating the normal radiographic appearance of OPG-based indices. DXA was not performed in these controls because, although it is a low-dose examination, it involves ionizing radiation which is not ethically justified in healthy young adults without a clinical indication.

The control group was therefore not intended to replace a DXA-negative validation cohort. Instead, it served as an ethically appropriate young-adult reference cohort for assessing normal classification behavior and potential false-positive classifications of the investigated OPG-based indices.

### 2.3. Outcomes

The primary outcomes were the practical applicability and the exploratory diagnostic performance of MOPS for identifying DXA-confirmed reduced bone status in the study group. Practical applicability was defined as the proportion of OPGs in which the index could be applied with sufficient visualization and image quality.

Secondary outcomes included comparison of MOPS with the established indices MCW, PMI, and MCI; normal classification rates in the young-adult reference control group; exploratory analyses; and non-applicability rates for each index under routine imaging conditions. These outcomes were selected to reflect the intended use of the method as an opportunistic pre-screening tool rather than as a stand-alone diagnostic test.

This investigation was planned as an exploratory retrospective pilot study rather than as a confirmatory diagnostic-accuracy trial. Consequently, no formal a priori sample-size calculation was performed. The sample size was determined by the number of eligible archive cases fulfilling all predefined criteria, particularly the availability of both OPG and DXA data within the permitted interval and sufficient image quality for index assessment. Of 45 potentially eligible cases with both OPG and DXA data, 40 remained after exclusion of cases with unusable DXA information. The 80-person control group was selected at a 2:1 ratio relative to the study group to improve the precision of normal classification rates while maintaining feasibility. All performance estimates should therefore be interpreted as exploratory and hypothesis-generating.

### 2.4. Eligibility Criteria and Image Acquisition

Inclusion criteria: To be included in the study group, patient records had to contain both an OPG and a DXA examination performed no more than 2 years earlier. In addition, the OPG had to depict the mandible and the C2-C3 region of the cervical spine sufficiently to allow image analysis.

For inclusion in the control group, participants had to be 20–30 years old, at least one OPG had to be available, no clinical or anamnestic evidence of osteoporosis had to be documented, and the radiograph had to depict the mandible and the C2-C3 region adequately for analysis. This age range was selected because it represents expected peak bone mass and is consistent with the young-adult reference principle underlying the WHO T-score definition.

Exclusion criteria: Patients were excluded if the mental foramina, the cortical bone of the inferior mandibular border, or the C2-C3 region were not adequately visible on the OPG. Further exclusion criteria were pathologies affecting the measurement region, such as tumors, cysts, or osteomyelitis, as well as documented current or previous use of osteoporosis medications or other drugs known to affect bone metabolism.

All panoramic radiographs were acquired independently of the study during routine clinical care by trained medical technical radiology assistants. The examinations were performed using the OP 3D Pro Ceph X-ray unit (KaVo Dental GmbH, Biberach, Germany) and the ProMax 2D system (Planmeca, Helsinki, Germany, Software: Planmeca Romexis 6.5.2). Standard patient positioning according to the manufacturer’s instructions was used [30].

DXA served as the reference standard in the study group. Bone mineral density (BMD) was assessed at the lumbar spine and/or proximal femur. T-scores were classified according to the World Health Organization (WHO) criteria: values of at least −1.0 were considered normal, values between −1.0 and −2.5 were classified as osteopenia, and values of −2.5 or lower were classified as osteoporosis [18,21].

The OPG-based indices were compared with the DXA-based classification in the study group. In the control group, the indices were compared with the presumed normal bone status of young adults without a documented history of osteoporosis.

### 2.5. Radiographic Index Assessment

Four indices (Figure 1) were evaluated independently by two examiners with different professional backgrounds, one in radiology and one in dentistry.

#### 2.5.1. Mandibular Cortical Width (MCW)

Mandibular Cortical Width (MCW), also known as the mental index, is a quantitative radiomorphometric parameter that reflects cortical thickness in the mandibular region adjacent to the mental foramen. It was defined as the vertical height of the mandibular cortex at the inferior border of the mandible caudal to the mental foramen, measured as the distance between the superior and inferior cortical margins (MCW = a, Figure 1) [30]. In accordance with previous reports, a diagnostic cutoff value of 3 mm was applied, with MCW values < 3 mm frequently interpreted as suggestive of reduced bone mineral density (BMD) [31,32,33].

#### 2.5.2. Panoramic Mandibular Index (PMI)

The panoramic mandibular index (PMI) is a dimensionless radiomorphometric ratio relating mandibular cortical thickness to the distance from the mental foramen to the inferior border of the mandible (Figure 1). In this study, PMI was calculated as a/b, where a represented the mandibular cortical thickness and b represented the distance from the inferior border of the mandible to the inferior margin of the visible mental foramen, measured along the perpendicular through the mental foramen to the tangent of the mandibular border [28,29]. Based on published thresholds, a cutoff value of 0.3 was used; values below 0.3 were considered pathological [34]. Further definitions of b for the PMI measurements are available in the literature [28,34,35,36,37].

#### 2.5.3. Mandibular Cortical Index; Klemetti Index (MCI)

The mandibular cortical index (MCI), also known as the Klemetti Index, is a qualitative parameter based on bilateral visual assessment of the inferior mandibular cortex distal to the mental foramen. Classification followed the criteria proposed by Klemetti and comprised three categories (Figure 1): C1, representing a normal cortex with a smooth and sharply defined endosteal margin, C2, indicating mild to moderate erosion with semilunar defects or endosteal cortical residues and C3, representing severe cortical erosion with pronounced porosity [29] (see Figure 1). This grading system has been widely applied in the literature as a screening indicator of cortical changes potentially associated with reduced skeletal bone status [29,33,38].

#### 2.5.4. Mini Osteoporosis Pre-Screening (MOPS)

In addition to the established radiomorphometric indices, the present study evaluated a qualitative three-stage classification, Mini Osteoporosis Pre-Screening (MOPS), based on visual assessment of the C2 and C3 cervical vertebral bodies.

MOPS was assessed using a predefined, stepwise visual protocol. First, the examining physician confirmed that at least one of these vertebral bodies was sufficiently pictured and not substantially obscured by positioning artifacts, superimposition, or motion blur. If neither C2 nor C3 could be evaluated with adequate confidence, MOPS was recorded as non-assessable.

Second, C2 and C3 were assessed separately. If both vertebral bodies were assessable, the vertebral body showing the most advanced osteoporosis-related appearance was selected for classification, provided that image quality was sufficient. This conservative rule was chosen by analogy with the clinical DXA principle that the most pathologically relevant measurement site determines the densitometric classification.

Third, classification was based on the overall radiographic appearance of the selected vertebral body, with particular attention to trabecular rarefaction, loss of non-load-bearing trabeculae, cortical accentuation, apparent cortical thinning, and the presence or absence of a vertebral body frame or “picture-frame” appearance. The index is intentionally treated as a qualitative screening marker; it is not used to diagnose osteoporosis or reliably distinguish osteopenia from osteoporosis.

Fourth, uncertain cases were graded according to the dominant visible pattern. A higher MOPS category was assigned only when the corresponding criteria were clearly visible; otherwise, the lower category was retained. This rule was used to reduce overclassification in routine images with imperfect positioning or incomplete cervical depiction [39,40].

Following the descriptive criteria reported by Dirisamer and Grampp (2002), cases were assigned to one of three categories [41] (Figure 2):

• MOPS 1 was assigned when the selected vertebral body showed no visible osteoporosis-related abnormalities, with preserved trabecular structure and no relevant cortical accentuation or picture-frame appearance (Figure 2a).
• MOPS 2 was assigned when early osteopenic or osteoporotic changes were visible, including rarefaction of non-load-bearing trabeculae, mild cortical accentuation, and a tendency toward a more homogeneous trabecular pattern without a clearly advanced picture-frame appearance (Figure 2b).
• MOPS 3 was assigned when advanced structural changes were present, including marked trabecular loss, pronounced cortical accentuation, apparent cortical thinning, and/or a distinct picture-frame appearance of the vertebral body (Figure 2c).

For all categories, MOPS was interpreted as an opportunistic radiographic warning sign. Grades 2 and 3 were considered suspicious for reduced bone status and, therefore, potentially relevant for referral for formal clinical and densitometric assessment, when consistent with the overall clinical context.

### 2.6. Image Quality Considerations and Statistical Analysis

Panoramic radiography was performed with a conventional field of view that included the maxilla, mandible, and adjacent skeletal structures, including the cervical spine up to approximately C3. Because panoramic imaging is strongly dependent on the focal trough, evaluation focused on whether the relevant anatomic regions were fully represented and whether positioning errors could have affected the visibility or geometry of the structures of interest [30].

Statistical analysis was performed descriptively using Microsoft Excel for Microsoft 365, Version 2512. The analysis followed the predefined outcome structure. For the primary outcome, MOPS applicability and exploratory diagnostic performance were assessed in the DXA-positive study group. For the secondary outcomes, MOPS was compared with MCW, PMI, and MCI; normal-classification rates were calculated in the young-adult reference control group; descriptive analyses were performed; and index-specific non-applicability rates were reported.

For each index, the number and percentage of assessable and non-assessable radiographs were calculated. Non-assessability was evaluated as a separate outcome because an index is only useful for opportunistic screening only if it can be applied reliably to routine images acquired without dedicated osteoporosis positioning protocols.

In the study group, DXA served as the reference standard. Because all study-group patients had DXA-confirmed osteopenia or osteoporosis, the analysis in this cohort primarily assessed each OPG-based index’s ability to detect reduced bone status among DXA-positive individuals. In the control group, OPG-based findings were compared with a presumed normal bone status, defined by young age, expected peak bone mass, and the absence of clinical or anamnestic evidence of osteoporosis (see WHO approach). Accordingly, results from the control group should be interpreted as normal classification rates and as exploratory estimates of specificity, rather than as definitive DXA-confirmed specificity values.

For each index, true-positive, false-negative, true-negative, and false-positive classifications were tabulated where applicable. Sensitivity was defined as the proportion of DXA-positive patients classified as osteopenic/osteoporotic by the respective OPG index. Specificity was defined exploratorily as the proportion of young presumed-normal controls who were classified as normal. Accuracy was calculated as the proportion of correctly classified individuals across the combined study and control groups. Failure rate was defined as the proportion of incorrect classifications relative to the applicable reference standard.

No confirmatory hypothesis testing was performed because the study was retrospective and exploratory and it had a limited sample size, particularly in the male subgroup. Sex-stratified analyses were therefore considered descriptive only. The statistical results should be used to evaluate feasibility, signal strength, and the design requirements for future prospective validation, not to claim definitive diagnostic validity.

## 3. Results

### 3.1. Study Population

A search of the RIS/PACS database identified 25,407 panoramic radiographs. Among these, 45 potential cases had both panoramic radiography and DXA data available for the study group. Five cases were excluded because the DXA data was unusable, leaving 40 patients for the final study-group analysis. The study group (Group 1) comprised 40 patients aged 27–90 years, including 34 women and 6 men. Female patients were 36–85 years old (mean age: 64 years), and male patients were 27–90 years old (mean age: 65 years). The control group (Group 2) included 80 young adults aged 20–30 years, comprising 40 women and 40 men. The mean age was 25 years for women and 23 years for men.

### 3.2. Applicability of the Radiographic Indices

The applicability of the four indices differed considerably in routine panoramic radiographs (Table 1). Among the 40 study-group radiographs, MCW could be assessed in 37 cases, PMI in 19, MCI in 40, and MOPS in 38. Thus, the proportion of non-assessable cases was lowest for MCI (0%) and MOPS (5%), whereas PMI showed the highest non-applicability rate (53%). MCW was not assessable in 8% of cases.

In the female study subgroup, PMI was not assessable in 19 of 34 cases (56%), whereas MCI was assessable in all cases and MOPS in 32 of 34 cases. In the male study subgroup, the corresponding numbers were limited by the very small sample size (N = 6) and should therefore be interpreted with caution.

### 3.3. Detection of Reduced Bone Status in the (DXA) Study Group

Overall, MCI and MOPS were the most consistently applicable indices in routine practice, whereas PMI showed limited practical applicability [35,36,37]. All patients in the study group had DXA findings consistent with osteopenia or osteoporosis. Detection rates in assessable cases were highest for MOPS and MCI and substantially lower for MCW and PMI (Table 1). No OPG-based index reliably differentiated osteoporosis from osteopenia in this dataset. Therefore, all OPG-based classifications were analyzed as normal versus osteopenic/osteoporotic for pre-screening purposes.

Compared with DXA, MOPS and MCI showed the highest agreement with the pathological bone status of the study group. These findings indicate that MCI and MOPS performed markedly better than MCW and PMI in identifying reduced bone status in DXA-positive patients. Because of its high non-applicability and limited discriminatory performance, PMI was not considered further in the subsequent comparative interpretation.

Across the indices, specificity was generally higher than sensitivity. Osteoporosis and osteopenia could not be reliably distinguished solely on the basis of OPG findings. OPG images for which an index could not be applied are shown in Table 2.

### 3.4. Normal Classification in the Young-Adult Reference Group

In the control group of young adults with presumed normal bone status, MCW and MOPS classified most cases as normal, whereas MCI classified a substantially lower proportion as normal. In this young-adult reference control group, selected according to the WHO young-adult peak-bone-mass concept and without clinical or anamnestic evidence of osteoporosis, MCW and MOPS classified most cases as normal, whereas MCI showed a substantially lower normal classification rate. Table 3 compares the different approaches with the WHO-based reference classification of normal, bone-healthy status in Group 2.

Overall descriptive accuracy across the combined study and reference groups was 66% for MCW, 63% for MCI, and 91% for MOPS. Sensitivity was 0.05 for MCW, 0.95 for MCI, and 1.00 for MOPS; exploratory specificity was 0.94, 0.48, and 0.86, respectively (Table 3).

### 3.5. Sex-Stratified Exploratory Analyses

Sex-stratified descriptive analyses were then performed for women (Table 4 and Table 5) and men (Table 6 and Table 7). These subgroup analyses were exploratory because the male DXA-positive subgroup was small.

Comparison of the different approaches with the WHO-based normal reference status among women in the control group (Table 5).

Comparison of the different approaches with DXA as the gold standard among men in the study group (Table 6).

Comparison of the different approaches with the WHO-based normal reference status among men in the control group (Table 7).

Sex-stratified analyses showed patterns generally similar to those observed in the overall cohort (Table 4, Table 5, Table 6 and Table 7). In women, descriptive accuracy was 53% for MCW, 74% for MCI, and 92% for MOPS; sensitivity was 0.06, 0.95, and 1.00, respectively, and exploratory specificity was 0.94, 0.58, and 0.90, respectively.

In men, MCI and MOPS classified all assessable DXA-positive cases as pathological, whereas MCW and PMI performed less consistently. Because only 6 men were included in the DXA-positive study group, these findings are descriptive and should be interpreted with caution.

### 3.6. Summary of Descriptive Findings

Table 8 summarizes the descriptive screening pattern of each index under routine imaging conditions and their use for incidental findings. The OPG images were done without special attention to the indices.

In summary, MCW showed high normal-classification rates in young adult controls but low detection rates in DXA-positive cases. MCI showed higher detection in DXA-positive cases but lower normal-classification rates in young-adult controls. PMI had the highest non-applicability rate. MOPS showed low non-applicability, high detection among assessable DXA-positive cases, and a high rate of normal classification in the young-adult reference group; these findings remain descriptive and require prospective validation.

## 4. Discussion

Several limitations restrict the interpretation of the results. First, this was a retrospective, single-center pilot study with a limited sample size. The male DXA-positive subgroup, in particular, was small; therefore, sex-specific analyses should be interpreted with caution. Second, the study group consisted exclusively of patients with DXA-confirmed osteopenia or osteoporosis. Consequently, the diagnostic accuracy compared to a DXA-negative control group of adults could not be fully determined relative to a DXA-negative adult control group. Third, no DXA examination was performed in the young-adult control group. This approach was considered ethically justifiable because DXA is not clinically indicated for healthy young adults without relevant risk factors. The age range of 20 to 30 years was chosen because this is when the expected maximum bone mass (BMDmax) is reached during this period, and this age range corresponds to the reference group for the WHO T-score definition. Nevertheless, the possibility of undetected pathological BMD in the control group cannot be ruled out in individual cases. Therefore, the results of the control group should be interpreted as an exploratory normal classification rather than a definitive DXA-confirmed classification. All panoramic radiographs were acquired under routine clinical conditions and not as part of a specific osteoporosis imaging protocol. Although this practice-oriented study design increases clinical relevance, it also introduces variation in positioning, field coverage, and image quality. Minor positioning corrections to improve cervical spine visualization may have affected the technical demands of quantitative indices such as MCW and PMI.

The jawbones exhibit high metabolic activity [42], and osteoporosis-related skeletal changes may therefore be visible on panoramic radiographs (OPGs). Radiological manifestations such as cortical thinning, particularly in the region of the mental foramen, and rarefaction or simplification of the trabecular structures can be analyzed [33,43]. Accordingly, OPGs have become increasingly important for detecting osteoporosis in recent years [33,44] and may serve as a preliminary screening tool to identify patients who could benefit from further DXA evaluation [45].

Radiological approaches can broadly be divided into quantitative and qualitative methods. Quantitative methods include the mandibular cortical width (MCW), the panoramic mandibular index (PMI), the computed tomography mandibular index (CTMI), the cone-beam mandibular index (CTI), and the A/M/P/S index. These methods are based on objectively standardized measurements of defined anatomical landmarks [33,46,47,48]. They require clear visualization of specific regions of interest (ROIs) and proper image acquisition. Projection, superimposition, and positioning errors can significantly distort the measurements [38]. The Mandibular Cortical Index (MCI) is a qualitative index based on the visual assessment of the inferior mandibular cortex according to the Klemetti classification [29,38]. The main advantages of the MCI are speed and simplicity. Its limitations include high subjectivity and low reproducibility, because the classification depends on the individual clinician’s experience. The lack of a metric scale also makes scientific comparability difficult [38].

The present findings are broadly consistent with previous reports. In a meta-analysis including 5266 women, Kinalski et al. (2020) reported a sensitivity of 0.81 and a specificity of 0.48 for osteopenia using MCI [33]. For osteoporosis, however, sensitivity decreased markedly to 0.35, whereas specificity remained 0.88 [33].

Similarly, Heuchert et al. (2024) emphasized the variability in visual MCI classification across examiners [42]. Calciolari et al. (2015) likewise reported limited reproducibility of MCI assessment [38]. Canal-Macías et al. (2025) demonstrated that severe cortical erosion (Klemetti C3 [49]) correlated significantly with reduced femoral-neck BMD [29,34].

For MCW, previous studies generally reported better diagnostic performance. Heuchert et al. (2024) described MCW as one of the most useful OPG-based markers, reporting a sensitivity of 0.712 and specificity of 0.804 using a threshold of ≤3 mm [42]. Similar conclusions were reported by Calciolari et al. (2015) and Kinalski et al. (2020) [33,38]. In the present study, however, MCW showed limited sensitivity in DXA-positive patients despite the favorable classification of healthy controls.

One possible explanation lies in the difference between controlled study conditions and routine clinical imaging. MCW depends on precise localization; even slight positional deviations can affect the projection of the target region, leading to superimposition or positional distortion of cortical thickness (e.g., slight caudal or cranial rotation). The present data therefore suggest that a quantitative marker may have limited utility if its measurement is not sufficiently robust in everyday panoramic radiographs. Similar limitations have been observed for PMI. Previous studies have reported sensitivities and specificities of approximately 0.7, depending on cutoff values and study design [33,38]. PMI is technically more demanding than MCW, which is reflected in the present study by its highest non-applicability rate. This result highlights an important limitation of many published studies, which often evaluate indices under idealized conditions, whereas opportunistic pre-screening in dental practice is based on routine X-rays.

The literature on OPG-based markers of osteoporosis is extensive but heterogeneous. Study populations, DXA protocols, reference thresholds, and statistical methods vary considerably across publications [33,44,46,50], making a direct comparison of diagnostic performance difficult. The present study therefore offers a pragmatic perspective by evaluating established indices in routine clinical rather than highly standardized research conditions. Against this background, MOPS appeared comparatively robust under routine clinical conditions. Unlike MCW and PMI, MOPS does not rely on technically demanding linear measurements at narrowly defined anatomical landmarks. Instead, it assesses the visual appearance of the C2 and C3 cervical vertebral bodies, including cortical accentuation, trabecular rarefaction, and the “picture-frame” appearance previously described in the literature. In the present study, this approach combined high applicability with a more balanced classification pattern than the established mandibular indices.

The present results are clinically relevant and consistent with the concept of opportunistic screening [45]. Panoramic radiographs are routinely acquired in dentistry for diagnostic, treatment-planning, implantological, and surgical evaluation purposes. They therefore offer the possibility of opportunistic osteoporosis screening without additional radiation exposure. From a practical perspective, a qualitative approach such as MOPS offers advantages in everyday clinical practice because it does not require technically demanding measurements and can be integrated quickly into standard diagnostic workflows. This is particularly relevant for patients with known osteoporosis risk factors, including postmenopausal women and older adults. In implantology and surgery, cortical thickness analysis can be a useful adjunct to diagnostics when digital volume tomography (DVT) is clinically indicated [47,51,52]. Further investigation may be necessary in cases of MOPS grade 2 or 3, mean cortical thickness (MCW) < 3 mm, or Klemetti class C3 [34,38]. However, abnormal radiographic findings should be communicated with caution and not interpreted as a definitive diagnosis. Dental radiographic findings can support interdisciplinary referral pathways but do not replace the established clinical and densitometric examination [45]. The dentist’s role is to assess risk and initiate further evaluation by a general practitioner or specialist when appropriate [45]. One possible approach could be similar to the AGSMO referral form, which is designed to structure interdisciplinary communication for patients treated with antiresorptive therapy [45].

This pilot study suggests that routine panoramic radiographs can facilitate opportunistic screening for osteoporosis in dental practice. The main finding of this exploratory study is that panoramic radiographs contain radiological information potentially relevant to opportunistic osteoporosis screening in routine clinical practice. Among the indices examined, MOPS demonstrated the best overall performance. It was applicable to most panoramic radiographs, identified all evaluable DXA-positive cases, and showed a high rate of normal findings in the young-adult reference group. These results support the hypothesis that assessment of the cervical vertebrae visible on panoramic radiographs can provide clinically relevant information beyond that obtained from conventional mandibular indices. The findings should be interpreted as evidence supporting early detection, not as a definitive diagnosis. MOPS was not intended to diagnose osteoporosis, replace DXA, or differentiate osteopenia from osteoporosis. Its potential role is limited to identifying patients with suspicious radiological findings who could benefit from further medical and densitometric evaluation. Future prospective studies should validate MOPS prospectively in larger, multicenter cohorts and systematically investigate whether patient positioning influences the screening markers in the mandible and cervical spine on panoramic radiographs. The need for training and standardized reports should also be emphasized. Geibel (2021) noted that the quality of radiographic interpretation in dental practice depends substantially on the clinician’s radiological training [53]. This is likely to be particularly true for subtle osteoporosis-related findings, which may be overlooked or overinterpreted depending on experience. Structured continuing education and clear reporting recommendations would therefore be useful if opportunistic osteoporosis prescreening is to be implemented more broadly. In the longer term, artificial intelligence (AI)-assisted tools may also support the standardized detection of potential cases of osteoporosis.

## 5. Conclusions

In summary, this pilot study supports the hypothesis that routinely acquired panoramic radiographs can contribute to opportunistic screening for osteoporosis in dental practice. As one of the most frequently used imaging techniques in dentistry, panoramic radiographs can reveal structural changes associated with osteoporosis. Among the methods investigated, the newly proposed Mini Osteoporosis Pre-Screening (MOPS) method demonstrated the most favorable ratio between applicability and practical discriminatory potential. Used as an opportunistic pre-screening tool, MOPS can assist trained dentists, orthodontists, and oral and maxillofacial surgeons in identifying potential osteoporosis-like changes and referring patients for further evaluation using basic screening or DXA. However, MOPS should not be regarded as a diagnostic test, a replacement for DXA, or a validated basis for clinical decision-making. The results of this pilot study should be investigated in larger prospective studies to confirm the reproducibility, predictive value, and clinical applicability of the approach before wider implementation is recommended.

## Figures and Tables

**Figure 1:**
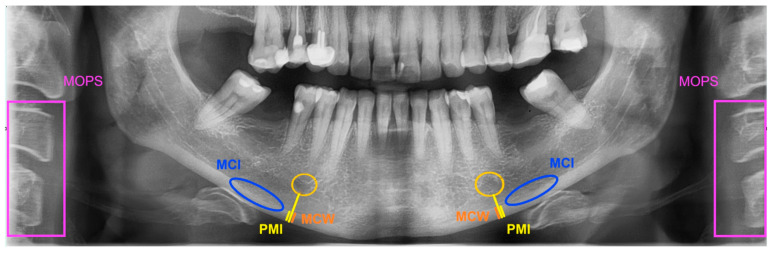
Panoramic radiograph of a patient with osteoporosis. The female patient is 54 years old (T-Score = −3). The different indices (MCW, PMI, MCI, MOPS) are schematically shown.

**Figure 2:**
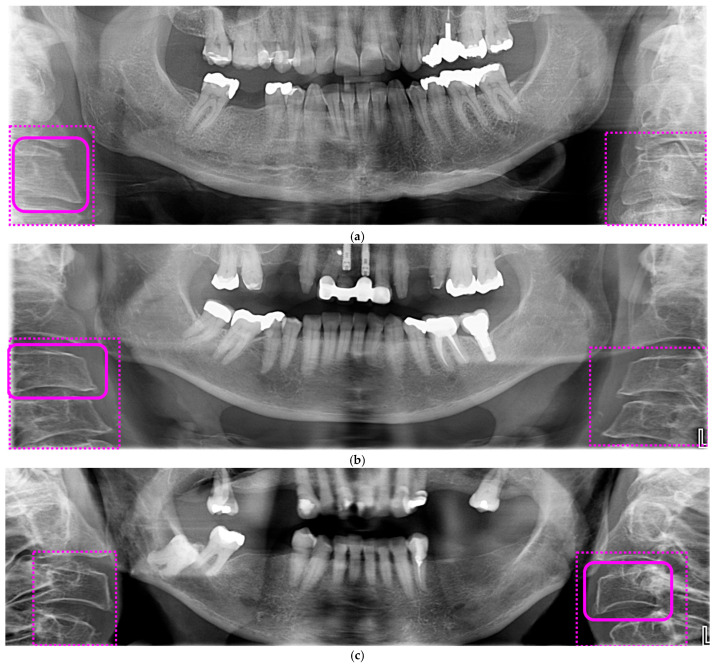
(**a**) Example panoramic radiograph for MOPS 1; female patient, 62 years old. (**b**) Example panoramic radiograph for MOPS 2; female patient, 54 years old, T-score = −2.1. (**c**) Example panoramic radiograph for MOPS 3; female patient, 73 years old, T-score = −3.0. The dotted line indicates the search area (cervical vertebral bodies C2 and C3), and the solid line marks the selected vertebral body.

**Table 1:** Applicability of the investigated indices relative to DXA in Group 1, comprising patients with osteoporosis or osteopenia. Diagnostic classifications for the respective indices are compared with DXA as the gold standard.

	DXA	MCW	PMI	MCI	MOPS
Total	40	37	19	40	38
osteoporosis or osteopenia	40	2	3	38	38
normal	0	35	16	2	0
correct classification rate	gold standard	5%	16%	95%	100%
misclassification rate/failure rate	gold standard	95%	84%	5%	0%

**Table 2:** Applicability of the investigated indices relative to DXA in Group 1, comprising patients with osteoporosis or osteopenia. Diagnostic classifications for the respective indices are compared with DXA as the gold standard by sex and index applicability.

	OPG Images	MCW	PMI	MCI	MOPS
all					
usable	40	37	19	40	38
not applicable	-	3	21	0	2
not applicable rate	-	8%	53%	0%	5%
women	34				
not applicable	-	0	19	0	2
not applicable rate	-	0%	56%	0%	6%
men	6				
not applicable	-	3	2	0	0
not applicable rate	-	50%	33%	0%	0%

**Table 3:** Comparison of the different approaches with the WHO-based normal reference status in the control group (Group 2), comprising young patients without a diagnosis of osteoporosis or osteopenia.

	WHO	MCW	MCI	MOPS
total	80	80	80	80
osteoporosis or osteopenia	0	5	42	11
normal	80	75	38	69
correct classification rate	reference standard	94%	48%	86%
misclassification rate/failure rate	reference standard	6%	53%	14%

**Table 4:** Comparison of the different approaches with DXA, the gold standard for the study group (Group 1), among women with DXA-confirmed osteoporosis or osteopenia.

	DXA	MCW	PMI	MCI	MOPS
Total	34	34	15	34	34
osteoporosis or osteopenia	34	2	3	32	32
Normal	0	32	12	2	0
correct classification rate	gold standard	6%	20%	94%	100%
misclassification rate/failure rate	gold standard	94%	80%	6%	0%

**Table 5:** Comparison of the different approaches with the WHO-based normal reference status in the control group (Group 2), women only, comprising young patients without a diagnosis of osteoporosis or osteopenia.

	WHO	MCW	MCI	MOPS
Total	40	40	40	40
osteoporosis or osteopenia	0	3	17	4
Normal	40	37	23	36
correct classification rate	reference standard	93%	58%	90%
misclassification rate/failure rate	reference standard	8%	43%	10%

**Table 6:** Comparison of the different approaches with DXA, the gold standard for the study group (Group 1), among men with DXA-confirmed osteoporosis or osteopenia.

	DXA	MCW	PMI	MCI	MOPS
Total	6	6	4	6	6
osteoporosis or osteopenia	6	0	0	6	6
normal	0	3	4	0	0
correct classification rate	gold standard	50%	0%	100%	100%
misclassification rate/failure rate	gold standard	50%	100%	0%	0%

**Table 7:** Comparison of the different approaches with the WHO-based normal reference status in the control group (Group 2), men only, comprising young patients without a diagnosis of osteoporosis or osteopenia.

	WHO	MCW	MCI	MOPS
Total	40	40	40	40
osteoporosis or osteopenia	0	2	15	7
normal	40	38	25	33
correct classification rate	reference standard	95%	38%	83%
misclassification rate/failure rate	reference standard	5%	63%	18%

**Table 8:** Comparison of the investigated indices with regard to the reliability of their screening results (“qualitative sensitivity”).

Diagnosis	MCW	PMI	MCI	MOPS
osteoporosis or osteopenia	no	no	yes	yes
normal	yes		no	yes

## Data Availability

The data presented in this study are available on request from the corresponding author since the supporting data can be found in hospital’s PACS system.

## Abbreviations

The following abbreviations are used in this manuscript:

AGSMO	Arbeitsgemeinschaft Supportive Maßnahmen in der Onkologie der Deutschen Krebsgesellschaft e.V.
AUC	Area Under the Curve
BMD	Bone Mineral Density
CBCT	Cone Beam Computed Tomography
CT	Computed Tomography
CTI	Cone Beam Mandibular Index
CTMI	Computed Tomography Mental Index
DXA	Dual-energy X-ray Absorptiometry
HU	Hounsfield Units
IOF	Internation osteoporosis foundation
MCI	Mandibular Cortical Index or Klemetti Index
MCW	Mandibular Cortical Width
MOPS	Mini Osteoporosis Pre-Screening
OPG	Orthopantomogram
PACS	Picture Archiving and Communication System
PMI	Panoramic Mandibular Index
RIS	Radiology Information System
ROI	Regions of interest
SD	Standard Deviations
SE	Sensitivity
SP	Specificity
WHO	World Health Organization
